# A mixed method study exploring gender differences in dementia caregiving

**DOI:** 10.1177/14713012231201595

**Published:** 2023-09-23

**Authors:** Vincent O. Poisson, Roslyn G. Poulos, Adrienne L. Withall, Ann Reilly, Leanne Emerson, Claire M. C. O’Connor

**Affiliations:** Dementia Australia, Australia; School of Population Health, 7800 The University of New South Wales, Australia; School of Population Health, The University of New South Wales, 7800 Australia; School of Population Health, The University of New South Wales, Australia; School of Psychology, The University of New South Wales, Australia; Dementia Australia, Australia; School of Population Health, 7800 The University of New South Wales, Australia; HammondCare, Centre for Positive Ageing, Australia; School of Psychology, 7800 The University of New South Wales, Australia; Neuroscience Research Australia, Australia

**Keywords:** male, carer, help-seeking, dementia, gender

## Abstract

Few studies have investigated the experience of male carers of people with dementia and fewer specifically examined whether male and female carers of people with dementia differ in their approach to the caring role. As such, this research set-out to investigate whether male carers of people with dementia approach the caring role differently to female carers. Data from 167 survey participants (24 males and 143 females) were analysed using a mixed research methodology. Participants’ demographics and scores on standardised burden and coping scales were analysed using linear regression. Participants’ written responses to open-ended questions were analysed using thematic analysis anchored in theories of hegemonic masculinity. No significant gender differences were identified in carers’ coping strategies or self-reported carer burden. However, qualitative analysis revealed strong thematic gender differences like: gendered barriers to help-seeking; gendered service preference; gendered considerations about residential care; gendered expression of burden; and themes of the absent son and exhausted daughter. This research identified that male carers of people with dementia approach help-seeking differently to female carers, typically focusing on addressing functional tasks and refraining from showing emotions, this despite reporting similar carer burden. Rapport building with male carers should start with conversations around functional issues rather than assessing the emotional impact of the caring role. The findings reinforce the need for more qualitative research into the unmet needs of male carers of people with dementia, to inform the design of male-friendly interventions which could facilitate timely access to services by male carers.

## Introduction

In 2022, up to 401,300 Australians were living with dementia and this number is expected to increase to 849,000 by 2058 (Australian Institute of Health and Welfare [AIHW], 2023; Dementia Australia [DA], 2023). Demographic estimates suggest that in 2022, 63% of people with dementia were female and 37% were male, with dementia being the leading cause of death in Australian females (AIHW, 2023; DA, 2023). Approximately 67% of people with dementia live in the community, of which 86% live in private dwellings and are being cared for by a spouse (50%) and/or an adult child (41%) (AIHW, 2023). With the projected increase in the number of people with dementia - and because dementia impacts more females than males - we may soon see an increase in male carers of people with dementia (Baker & Robertson, 2008; Geiger et al., 2015; Greenwood & Smith, 2015; Pöysti et al., 2012; Robinson et al., 2014). However, research reviews across the caregiving literature have highlighted that there is a paucity of research on male carers of people with dementia - as a specific cohort – with most research studies adopting a broad brush stroke and reporting on the experience of males who identify as carers “in general” and/or carers of a person with a long-term chronic illness (Baker & Robertson, 2008; Fee et al., 2020; Grigorovich et al., 2016; Houde, 2002; McDonnell & Ryan, 2013, p.238; McDonnell & Ryan, 2014; Robinson et al., 2014).

### The male approach to the caring role

Historically, caregiving was socially and politically constructed as a “feminised activity” (Baker et al., 2010, p.319) and research suggests that males typically struggled with the adaptation from “being a man, to becoming a caring man” (Hellström et al., 2017, p.958). According to O’Neil’s (1981a, 1981b) gender role conflict theory, for males, the caring role triggers a fear of being perceived by others as feminine, consequently leading to a psychological conflict with their self-identity and reinforcing poor help-seeking behaviour. Connell’s (1995) hegemonic masculinity theory also proposes that society reinforces stereotypical male behaviour, encouraging men to distance themselves from nurturing roles because these are incompatible to “the honoured way of being a man”, which could explain why male carers approach the caring role differently to female carers (Connell & Messerschmidt, 2005, p.832).

It has also been suggested that males approach the caring role as a job, using task-focused strategies to deal with the challenges of the caring role (Black et al., 2009), whereas females adopt emotion-focused coping styles (Calasanti & King, 2007; Geiger et al., 2015; Zarit et al., 1986). Additionally, males have smaller social networks and are more socially isolated than females (Dam et al., 2017; Fee et al., 2021). According to the literature, males will typically reject the need for social support, because they want to preserve the image of being “an independent guy” (McKenzie et al., 2018, p.1251) and it is argued that this conformity with masculine norms increases their risk of developing mental and physical health problems (Herreen et al., 2021).

Research suggests that boys have been socialised to be brave and to tolerate painful experiences, whilst girls have been socialised to show emotions and verbalise discomfort, suggesting that gender-role expectations shape people’s embodiment and report of strain (Myers et al., 2003; Samulowitz et al., 2018). Whilst female carers have been reported to experience higher levels of carer strain than male carers (Brodaty et al., 2014; Greenwood & Smith, 2015; Robinson et al., 2014; Zarit et al., 1986), the “Systemic Response Bias” theory suggests that to maintain their masculinity, male spousal carers might adopt a defensive response bias (O’Rouke et al., 1996, p.378), only reporting the amount of burden that they are “willing to report”, because otherwise, any admission about failure to manage would imply a sense of weakness and/or that they might not be “man enough” to complete their roles as husbands (Baker et al., 2010, p.325). Male carers might experience equally high levels of carer burden but it might be socially desirable for them to report low burden (Baker et al., 2010; O’Rourke et al., 1996).

Male carers have been described as “ineffective” in their approach to the caring role (Russell, 2001, p.353) because they tend to normalise the cognitive changes of dementia (Hayes et al., 2010) and are less likely to identify as “the carer”, which could explain their poor access to formal supports (Greenwood & Smith, 2015; Milligan & Morbey, 2013, p.20). Compared to female carers, males will wait for a crisis to happen before asking for help (McDonnell & Ryan, 2013; Milligan & Morbey, 2016) and will more readily admit the person into hospital and/or residential care (McDonnell & Ryan, 2014; Robinson et al., 2014).

### Knowledge gap

Despite the increasing dependence on males to care for a person living with dementia, only a small amount of research has specifically examined how male carers of people with dementia cope with the demands of the caring role in comparison to female carers (Greenwood & Smith, 2015; Kokorelias et al., 2021; Pöysti et al., 2012; Robinson et al., 2014). Recently, an analysis of service access data suggested that male carers were under-referred to, and underutilised, services offered by an Australian peak body organisation providing advice, counselling and education to all people impacted by dementia (Poisson et al., 2020). Indeed, earlier research identified that many carer support services are not ‘man-friendly’ and are primarily set-up to support women (Malcher, 2005; Milligan & Morbey, 2013; Ricciardelli et al., 2012; Sanders & McFarland, 2002). A policy statement by Carers Australia NSW (2016) reported that males are often unrecognised as carers by health agencies because these agencies have historically serviced a female client-base, which might have led to the unintended exclusion of male carers.

This study aimed to explore gender in the context of dementia caregiving, by investigating whether: (a) male carers were less likely than female carers to identify as ‘the carer’, (b) male carers approached the care duties with a different coping style, in comparison to female carers, (c) male carers reported differing levels of carer burden, in comparison to female carers, and (d) gender differences exist in how carers appraised and accessed formal support services.

## Method

### Study design

We applied a mixed research methodology, with a concurrent embedded design: experimental model (Creswell & Plano Clark, 2006), using an online survey to capture qualitative and quantitative data at the same time. Quantitative data were sourced from the participants’ demographics and scores on two standardised psychometric measures intended to address the main research questions guiding this study. Qualitative data came from participants’ written responses to a series of open-ended questions, anchored in Connell’s (1995) hegemonic masculinity theory and formatted in a way to facilitate a richer exploration of gender in the context of how carers of people with dementia approached the caring role (Connell & Messerschmidt, 2005).

### Participants and recruitment

Participants voluntarily took part in an online survey posted on the research webpage of an Australian dementia peak body organisation. Participants were eligible if they were aged 18 years or above, living in Australia, were the main support person for the person with dementia and provided care without any monetary agreement. One of our main research questions was to determine whether male participants were less likely to identify themselves as ‘a carer’, therefore the term ‘main support person’ was instead advertised in our eligibility criteria. However, for the purposes of reporting this research, we hereon in will describe the participants as ‘carers’ if they matched the above eligibility criteria.

Studies investigating the experience of male carers of people with dementia have mostly been qualitative and/or recruited sample sizes varying from 15 to 100 participants (Baker & Robertson, 2008; McDonnell & Ryan, 2013; Pöysti et al., 2012; Robinson et al., 2014). Research suggests that male carers underutilise carer support services (Poisson et al., 2020) and are less likely to identify as a carer; therefore, we anticipated the population of male carers of people with dementia to be small and difficult-to-recruit (Greenwood & Smith, 2015; Milligan & Morbey, 2013). Dementia carer population estimates in Australia are inconsistent (AIHW, 2023) and this study was unfunded, targeted a hard-to-reach population, and was designed to be exploratory. As such, a non-probability-based purposive sampling strategy was applied and no limits were placed on the sample size (Liamputtong, 2020, p.17; Raifman et al., 2022). However, the survey remained open for a period of three months to maximise the sample size.

### Instruments

The online survey used *Survey Monkey* software, and collected participant demographic information such as relationship to the person with dementia, whether they identified as ‘a carer’ for the person with dementia, gender, age, language spoken at home, employment status, whether or not they identified as Aboriginal and/or Torres Strait Islander, hours of support provided in a week, duration of caring role, and information about the person with dementia (e.g., dementia diagnosis, gender, age and living arrangement). The survey included standardised psychometric tools to measure coping (using The Coping Strategy Indicator (CSI) scale) (Amirkhan, 1990, 1994) and burden (using the 12-Item Zarit Burden Interview (ZBI)) (Lin et al., 2017), and also included a series of open-ended questions to capture the more subjective aspects of our research.

#### Coping strategy

The Coping Strategy Indicator (CSI) scale (Amirkhan, 1990, 1994) was applied to test whether male participants used different coping styles to female participants. The CSI is a 33 item, 3-point self-report scale designed to assess basic modes of coping over three scales: scale 1 (Problem solving), scale 2 (Seeking social support) and scale 3 (Avoidance) (Amirkhan, 1990, 1994). The CSI has significant generalisability across populations and its scales indicate high internal reliability (with Cronbach’s alpha coefficients of .84 (Avoidance), .89 (Problem Solving), and .93 (Seeking Support)) (Amirkhan, 1990, 1994; Desmond et al., 2006).

#### Carer burden

The 12-Item Zarit Burden Interview (ZBI) assessed carer strain. The precision of the 12-item ZBI is equivalent to the original 22-item ZBI, with internal consistency and concurrent validity found to be satisfactory to provide an accurate assessment of carer burden between genders and should take less than 10 minutes to complete (Lin et al., 2017).

#### Open-ended questions

These questions were formulated based on Connell’s (1995) hegemonic masculinity theory, the available literature on how male carers approach the caring role, and the clinical expertise of the research team. The questions, which were piloted with a consumer advocate from an Australian dementia peak body organisation, aimed to capture the participants’ perceptions about how well (or not) formal support services were addressing their needs (e.g., (i) *Which service has been the most useful for you, and why?* (ii) *Which service has been the least useful for you, and why?* (iii) *What changes would you make to the services you receive (if any) to help them better meet your needs?*) and what would motivate participants to seek help from formal support services (e.g., *Which circumstances or events would trigger you to ask for help from services in order to better support the person with dementia?*). The full list of open-ended questions is detailed in Appendix 1.

### Quantitative data analysis

Statistical analyses were conducted using the R statistical software package. Residual plots showed that outcome variables were normally distributed with constant variance. Therefore, linear regression were applied to examine the effect of the carer’s gender on the CSI (Scale 1, Scale 2 and Scale 3) and level of burden (ZBI), while controlling for the effect of potential confounding variables (i.e., carer’s relationship to the person with dementia; participant’s identity as a carer; carer’s age; carer’s employment status; diagnosis of the person with dementia; gender of person with dementia; age of person with dementia; living arrangement of the person with dementia; number of care hours provided by the carer; and length of time spent in the caring role). The Holm-Bonferroni method (Holm, 1979) was applied, that is, we adjusted the *p*-values (from lowest to highest) for 44 tests performed, to control the family-wise error rate (probability of any false positives) due to multiple testing.

### Qualitative data analysis

Thematic analysis, based on Braun and Clarke’s (2006) 6-steps framework, was used to analyse participants’ written responses to a series of open-ended questions and to identify core thematic differences between how male and female participants approached different dimensions of the caring role. This framework was chosen for its proven validity and reliability in similar research contexts and most importantly because the guidelines provided enough flexibility to be adapted in applied research settings (Braun & Clarke, 2021; Maguire & Delahunt, 2017; Nowell et al., 2017). The analysis was driven by the research questions and the available literature on how male carers approach the caring role (Baker & Robertson, 2008; Fee et al., 2020, 2021; Greenwood & Smith, 2015; Houde, 2002; McDonnell & Ryan, 2013; Milligan & Morbey, 2013; Robinson et al., 2014; Sharma et al., 2016). The analysis was undertaken by the first author, with support from the co-authors.

Familiarisation with the data involved reading all the participant responses to form an early impression of the depth and breadth of the entire “data corpus” (Maguire & Delahunt, 2017, p.3355). The deductive analysis was driven by the main questions guiding this research and most of the codes were “theory driven” as they matched elements of the literature (Braun & Clarke, 2006, p. 88). Once all the data was coded and collated, themes that emerged from the data were generated through a process of comparing codes between different carer relationship groups (i.e., wives/female partner, daughters, other females, husband/male partners, sons, and other males). The first and last author then reviewed and defined the themes. Verbatim extracts were included to provide an “authentic narrative argument”, directly aimed at answering the research questions (Braun & Clarke, 2006, p. 93). Participants’ quotes were de-identified using a unique identification number. For example, a male (M) who identified as husband (H), would be identified as: MH###.

## Results

### Sample characteristics

The sample comprised of 167 participant entries and their demographic data are illustrated in Table 1

**Table 1.** Demographic variables describing the sample (*n* = 167).

	Male	%	Female	%	Total	%
	*n* = 24	14	*n* = 143	86	*n* = 167	100
Participants' relationship to person with dementia						
Spouse/Partner	18	75.00	79	55.24	97	58.08
Adult child	3	12.50	54	37.76	57	34.13
Other	2	8.33	10	6.99	12	7.19
Brother/Sister-in-law	1	4.17	0	.00	1	.60
Participants who identified as ‘a carer’						
Yes	22	91.67	134	93.71	156	93.41
No	0	.00	2	1.40	2	1.20
Unsure	1	4.17	6	4.20	7	4.19
Other	1	4.17	1	.70	2	1.20
Participants’ age group (years)						
Under 40 years	0	.00	5	3.50	5	2.99
40–54 years	3	12.50	20	13.99	23	13.77
55–69 years	10	41.67	72	50.35	82	49.10
70–84 years	10	41.67	43	30.07	53	31.74
Above 85 years	1	4.17	3	2.10	4	2.40
Participants’ employment status						
Full-time	3	12.50	19	13.29	22	13.17
Part-time	2	8.33	30	20.98	32	19.16
Retired	15	62.50	69	48.25	84	50.30
Not employed	4	16.67	21	14.69	25	14.97
Studying	0	.00	4	2.80	4	2.40
Diagnosis of person with dementia						
Alzheimer’s disease	13	54.17	68	47.55	81	48.50
Vascular dementia	2	8.33	21	14.69	23	13.77
Frontotemporal dementia	4	16.67	14	9.79	18	10.78
Not sure	1	4.17	11	7.69	12	7.19
Other	1	4.17	14	9.79	15	8.98
Mixed	3	12.50	15	10.49	18	10.78
Gender of person with dementia						
Female	19	79.17	50	34.97	69	41.32
Male	5	20.83	93	65.03	98	58.68
Age of person with dementia						
Under 40 years	0	.00	0	.00	0	.00
40–54 years	0	.00	3	2.10	3	1.80
55–69 years	10	41.67	26	18.18	36	21.56
70–84 years	10	41.67	74	51.75	84	50.30
85 years and over	4	16.67	40	27.97	44	26.35
Living arrangement of person with dementia						
They live with me	21	87.50	111	77.62	132	79.04
They live on their own	1	4.17	20	13.99	21	12.57
They live with relatives or other person	2	8.33	10	6.99	12	7.19
Other	0	.00	2	1.40	2	1.20
Hours spent caring for person with dementia						
Less than 10 hours	1	4.17	5	3.50	6	3.59
10–15 hours	1	4.17	22	15.38	23	13.77
15–30 hours	3	12.50	21	14.69	24	14.37
30–40 hours	2	8.33	18	12.59	20	11.98
More than 40 hours	17	70.83	77	53.85	94	56.29
Period spent caring for person with dementia						
Less than 6 months	1	4.17	4	2.80	5	2.99
6 months - 2 years	4	16.67	33	23.08	37	22.16
2 years–5 years	9	37.50	63	44.06	72	43.11
5 years–10 years	10	41.67	32	22.38	42	25.15
More than 10 years	0	.00	11	7.69	11	6.59
Has carer already initiated access to services?						
Yes	17	70.83	102	71.33	119	71.26
No	5	20.83	37	25.87	42	25.15
Did not answer	2	8.33	4	2.80	6	3.59
Did carer perceive they had received enough services						
Yes	3	12.50	15	10.49	18	10.78
No	8	33.33	47	32.87	55	32.93
Unsure	5	20.83	27	18.88	32	19.16
Did not answer	8	33.33	54	37.76	62	37.13
Did carer perceive that services had met their needs						
Yes	5	20.83	28	19.58	33	19.76
No	5	20.83	41	28.67	46	27.54
Unsure	6	25.00	20	13.99	26	15.57
Did not answer	8	33.33	54	37.76	62	37.13

. In brief, a majority (86%) of participants identified as female: 55% of females identified as a wife/female partner and 38% daughters of the person with dementia. Males accounted for 14% of participants, of whom 75% identified as husbands/partner, 13% as sons and the remainder as ‘other’ or ‘brother-in-Law’. Most (93%) participants identified as ‘a carer’ and the remainder identified as either: ‘no’ [not a carer] (1%); ‘unsure’ (4%); or as ‘other’ (1%). Only those who identified as ‘Other’ chose to expand on their identity and the nature of their relationship to the person with dementia. These comments were used to determine whether participants fit within the study inclusion criteria. For example, a female wrote: “wife in the first instance” (FW29) and a male wrote: “close friend…always on call for him and family” (MO3); these comments suggested that these participants matched the eligibility criteria and therefore, included in the sample. Participants who identified as ‘Other’ and whose comments indicated that they did not match the eligibility criteria, were excluded from the sample prior to analysis; for example, a female wrote: “Facilitator to a program…for consumer who resides in the nursing home” (FO6). Approximately half (49%) of participants were aged between 55 to 69 years and 50% were retired, but some (13%) were still employed full-time. Many (56%) participants provided more than 40 hours of direct care per week and 79% were living in the same house as the person with dementia. The largest proportion of males (42%) had been in the caring role for 5–10 years, while the largest proportion of females (44%) had spent 2–5 years in the caring role. Additionally, 80% of females versus 58% of males cared for a person aged above 70 years, whilst 42% of males versus 18% of females, cared for a person aged between 55 to 69 years. Almost 71% of participants had accessed formal services.

### Quantitative results

#### Coping Strategy Indicator (CSI)

A proportion (21%) of the CSI data were invalid (e.g., missing data) and 132 (n (female) = 114: n (male) = 18) responses were included in the analysis. Table 2

**Table 2.** Mean and standard deviation scores on the CSI and ZBI.

	Coping Strategy Indicator (CSI) scale								12-Item Zarit Burden Interview (ZBI) scale			
	n	%	Mean (standard deviation) scores						n	%	Mean (standard deviation) scores	
			Scale 1 problem solving		Scale 2 seeking social support		Scale 3 avoidance					
Husband/Partner	13	72.22	21.77	(5.09)	17.77	(5.07)	19.08	(5.20)	16	72.73	26.88	(7.01)
Son	3	16.67	28.67	(2.08)	21.00	(.00)	23.67	(5.77)	3	13.64	32.00	(6.56)
Other male	2	11.11	24.00	(7.07)	25.00	(2.83)	18.00	(4.24)	2	9.09	12.00	(2.83)
Brother-in-law	0	.00							1	4.55	30.00	
Males (total)	18	100	23.17	(5.35)	19.11	(4.97)	19.72	(5.24)	22	100	26.36	(8.04)
Wife/Partner	69	60.53	24.88	(5.27)	21.42	(4.32)	19.14	(4.24)	77	55.40	28.16	(8.34)
Daughter	40	35.09	26.88	(5.54)	22.25	(4.62)	18.48	(4.49)	52	37.41	26.54	(7.26)
Other female	5	4.38	25.60	(4.56)	23.00	(5.70)	19.40	(3.78)	10	7.19	22.30	(10.12)
Sister-in-law	0	.00							0	.00		
Females (total)	114	100	25.61	(5.38)	21.78	(4.47)	18.92	(4.29)	139	100	27.13	(8.18)
Total (males & females)	132								161			

shows scores for all included males and females, and scores by relationship to person with dementia. Table 3

**Table 3.** Regression analyses: Carer gender as a predictor of scores on the 12-item Zarit Burden Interview (ZBI: *n* = 161) and Coping Strategy Indicator (CSI: *n* = 132).

Dependent variables	Df	*β*	95% CI	*R* ^2^	F	Raw *p*-value	Holm-Bonferroni adjusted *p*-values
Primary analysis: Effect of carer’s gender on ZBI and CSI scores (adjusting for confounding variables)							
ZBI scores	1, 125	−1.811	−6.482, 2.860	.139	.589	.444	1.000
CSI Scale 1 (problem solving)	1, 97	−2.388	−5.929, 1.153	.091	1.792	.184	1.000
CSI Scale 2 (seeking social support)	1, 97	−3.550	−6.783, −.317	−.046	4.750	.032	.349
CSI Scale 3 (avoidance)	1, 97	1.106	−1.778, 3.990	.093	.579	.449	1.000
Secondary analysis: Effect of individual confounding variables on ZBI and CSI scores							
1. Effect of carer’s relationship to person with dementia							
ZBI scores	3, 157	1.099	−1.584, 3.782	.037	3.062	.030	.239
CSI Scale 1 (problem solving)	2, 129	−2.610	−4.592, −.628	.035	3.395	.037	.402
CSI Scale 2 (seeking social support)	2, 129	−1.321	−3.026, .384	.015	1.995	.140	1.000
CSI Scale 3 (avoidance)	2, 129	.297	−1.360, 1.954	−.015	.063	.939	1.000
2. Effect of participant’s identity as carer							
ZBI scores	3, 157	6.753	−4.716, 18.223	−.005	.752	.523	1.000
CSI Scale 1 (problem solving)	3, 128	−6.732	−17.550, 4.087	−.010	.551	.649	1.000
CSI Scale 2 (seeking social support)	3, 128	.276	−8.872, 9.425	.003	1.132	.339	1.000
CSI Scale 3 (avoidance)	3, 128	6.130	−2.604, 14.864	.009	1.379	.252	1.000
3. Effect of carer’s age (years)							
ZBI scores	4, 156	−2.304	−10.295, 5.687	−.015	.418	.796	1.000
CSI Scale 1 (problem solving)	4, 127	−8.842	−16.753, −.931	.015	1.486	.210	1.000
CSI Scale 2 (seeking social support)	4, 127	−1.842	−8.707, 5.023	−.024	.232	.920	1.000
CSI Scale 3 (avoidance)	4, 127	1.737	−4.744, 8.217	.005	1.150	.336	1.000
4. Effect of carer’s employment status							
ZBI scores	4, 156	−.659	−9.494, 8.175	−.022	.134	.970	1.000
CSI Scale 1 (problem solving)	4, 127	−11.722	−22.493, −.951	.044	2.492	.046	.463
CSI Scale 2 (seeking social support)	4, 127	1.778	−7.561, 11.117	.008	1.261	.289	1.000
CSI Scale 3 (avoidance)	4, 127	12.444	3.762, 21.127	.065	3.256	.014	.154
5. Effect of diagnosis of person with dementia							
ZBI scores	5, 155	−1.398	−5.336, 2.539	−.023	.273	.927	1.000
CSI Scale 1 (problem solving)	5, 126	1.288	−1.722, 4.297	−.005	.859	.511	1.000
CSI Scale 2 (seeking social support)	5, 126	.983	−1.604, 3.570	−.025	.352	.880	1.000
CSI Scale 3 (avoidance)	5, 126	.191	−2.268, 2.649	−.010	.742	.593	1.000
6. Effect of gender of person with dementia							
ZBI scores	1, 159	1.409	−1.171, 3.990	.001	1.163	.283	1.000
CSI Scale 1 (problem solving)	1, 130	−1.030	−2.952, .892	.001	1.123	.291	1.000
CSI Scale 2 (seeking social support)	1, 130	.220	−1.423, 1.863	−.007	.070	.792	1.000
CSI Scale 3 (avoidance)	1, 130	.500	−1.072, 2.071	−.005	.396	.530	1.000
7. Effect of age of person with dementia							
ZBI scores	3, 157	−.439	−10.082, 9.204	−.006	.684	.563	1.000
CSI Scale 1 (problem solving)	3, 128	3.425	−2.918, 9.767	.037	2.691	.049	.463
CSI Scale 2 (seeking social support)	3, 128	2.424	−3.060, 7.908	.007	1.302	.277	1.000
CSI Scale 3 (avoidance)	3, 128	.182	−5.114, 5.478	−.011	.546	.652	1.000
8. Effect of living arrangement of person with dementia							
ZBI scores	3, 157	8.087	−3.090, 19.262	.048	3.711	.013	.116
CSI Scale 1 (problem solving)	3, 128	−.528	−8.202, 7.146	−.007	.720	.542	1.000
CSI Scale 2 (seeking social support)	3, 128	1.620	−4.874, 8.115	.005	1.230	.302	1.000
CSI Scale 3 (avoidance)	3, 128	−1.454	−7.749, 4.841	−.019	.168	.918	1.000
9. Effect of hours spent providing care to person with dementia							
ZBI scores	4, 156	3.767	.224, 7.309	.110	5.964	.000	.002
CSI Scale 1 (problem solving)	4, 127	1.756	−1.144, 4.656	−.017	.460	.765	1.000
CSI Scale 2 (seeking social support)	4, 127	.389	−2.866, 2.089	−.024	.231	.920	1.000
CSI Scale 3 (avoidance)	4, 127	.396	−1.909, 2.702	.033	2.108	.084	.836
10. Effect of years spent providing care to person with dementia							
ZBI scores	4, 156	−3.662	−9.093, 1.769	.088	4.872	.001	.010
CSI Scale 1 (problem solving)	4, 127	.483	−3.561, 4.528	−.005	.843	.501	1.000
CSI Scale 2 (seeking social support)	4, 127	.283	−3.172, 3.738	−.012	.617	.651	1.000
CSI Scale 3 (avoidance)	4, 127	.975	−2.319, 4.269	−.003	.889	.473	1.000

ZBI - Zarit Burden Interview. CSI - Coping Strategy Indicator.

shows that after performing a linear regression controlling for potential confounding variables and then controlling the *p*-value for multiple testing using the Holm-Bonferroni method, we found no differences between gender on the CSI for scale 1 (Problem Solving Strategy; F (1,97) = 1.792, *p* = .184, Holm Adj *p* = 1.000, *R*^2^ = .091); scale 2 (Seeking Social Support Strategy; F (1,97) = 4.750, *p* = .032, Holm Adj *p* = .349, *R*^2^ = −.046), or scale 3 (Avoidance Strategy; F (1,97) = .579, *p* = .449, Holm Adj *p* = 1.000, *R*^2^ = .093).

#### The 12-item Zarit Burden Interview (ZBI)

A proportion (4%) of the ZBI data were invalid (e.g., missing data) and 161 (*n* = 139 female: *n* = 22 male) responses were included in the analysis. Table 2 shows scores for all included males and females, and scores by relationship to person with dementia. Table 3 shows that after performing a linear regression controlling for potential confounding variables and then controlling for multiple testing using the Holm-Bonferroni method, we found no evidence (F (1,125) = .589, *p* = .444, Holm Adj *p* = 1.000, *R*^*2*^ = .139) of an effect of the carer’s gender on ZBI scores (*β* = −1.811, 95% CI: −6.482, 2.860).

### Qualitative results

In contrast to the quantitative analysis, our qualitative data revealed gender differences for carers of people with dementia. Gendered responses were seen across five overarching themes, as listed in Table 4, which also includes extracts of the participants’ written comments to illustrate gendered differences.

**Table 4.** Thematic gender differences in carers of people with dementia.

	Males	Females
Gendered barriers to help-seeking	Wait until their person with dementia gives permission to ask for help	Wait until the care needs of their person with dementia exceeds their caring capacity
	“If my wife asked me or we could discuss it”*(*MH15)	“Progression in my husband that I can no longer support to the best of my ability” (FW56)
Gendered service preference	Home help to spend more time with the person with dementia	Respite to get more time away from the person with dementia
	“Carer comes out to assist with cooking cleaning and also looking after my wife.” (MH4)	“Day club respite gives me a chance to be relieved of responsibility temporarily” (FW2)
	“Cleaning bathroom and floors, one less task for myself” (MH17)	
Gendered consideration about residential care	When their own health becomes at risk	When their own safety becomes at risk
	“If I was drowning in a sea of impossibilities and demands that were severely hurting my own health” (MH13)	“If he got violent and I didn’t feel safe” (FW67)
Gendered expression of burden	Functional burden	Emotional burden
	“Struggling to communicate. Lots of misunderstandings” (MH13)	“It’s the emotional loss that creates the despair” (FW20)
The absent son and exhausted daughter	Manage and delegate from a distance	Fully immersed in the caring role
	“Is there more help out there?” (MS2)	“Having to always be ‘on call’… Not having any time to me” (FD14)

#### Gendered barriers to help-seeking

Approximately 25% of participants in this study (male 21% and female 26%) confirmed they had not yet accessed services at the time of completing this survey.

##### Males: If my wife asked me

Male participants expressed finding it “hard to access” (MH15) services because the person with dementia denied having dementia or was “extremely secretive about” (MH18) their diagnosis and therefore, male carers said that they would consider accessing services only if, or when, “my wife asked me, or we could discuss it” (MH15). Exemplified here:

My wife resists it sometimes and says she can look after herself…in my situation I don't have any real control in what to do. It depends how my wife feels, and this can change. The first rule of dementia is don't argue. This is hard but it is better than the other option. (MH5)

##### Females: I can manage on my own

Most female participants felt that they were “not up to that stage” (FW66) because the symptoms of dementia were “not severe enough” (FW41) and therefore, “all things were manageable” (FW6) and they were “coping at the moment” (FW65). Exemplified here:

I don’t feel my husband needs support services as yet…and would consider asking for help if I can no longer support him to the best of my ability. (FW56)

#### Gendered service preference

##### Males: Domestic support services

Male participants articulated a strong need for domestic cleaning services, as these reduced their workload, writing comments like: “Cleaning bathroom and floors, one less task for myself” (MH17) which created the opportunity for them to spend more time with their wives. Males described residential respite as “a disaster” (MH6) and a “double-edge sword” (MH5), because of “too much stress involved” (MH3) in organising residential respite services, saying “I am too exhausted to spend time planning” (MH11) and “my care receiver prefers to be with me and she enjoys my company and support best” (MH3).

##### Females: Respite services

Female participants however, seemed to articulate a strong need for more day respite hours, as it gave them “a chance to be relieved of responsibility temporarily” (FW2) and it provided “some relief from chores - enabling self-care” (FW26). Many spoke highly of respite, comparing it to “time off” (FW30), and an opportunity “to do the things I really enjoy” (FW50).

#### Gendered considerations about when to access residential care

Both genders shared a view that residential care was not their preferred option and was described as a last resort by most. However, we identified gender differences in the reasons and/or circumstances that would push carers to eventually consider placing the person with dementia into residential care.

##### Males: Their own health

Most male participants said that they would consider accessing residential care, “only as the last resort” (MH3), if and when, the person with dementia presented with care needs which would impact on their own health. Exemplified here:

If my spouse got to the point where she was needing much more than I could provide and was behaving in ways that were difficult to handle. For example, walking and getting lost. Struggling to feed herself, clean herself, dress herself. Was totally dependent on me for her survival. And in the middle of it, I found like I was drowning in a sea of impossibilities and demands that were severely hurting my own health. (MH13)

##### Females: Violence

Female participants strongly identified violence and physical aggression from the person with dementia, as their main trigger to accessing residential care services. For example: “If he got violent and I didn’t feel safe, but it would be an absolute final decision as I do not want him to go there.” (FW67)

#### Gendered expressions of burden

##### Males: Functional burden

Male participants applied a functional language to describe their caring engagement, for example, saying that they were “working” (MH4) with “a bit of a unique case” (MH1) and found the caring role to be “a learning experience” (MH1). A participant described their person with dementia as their “care receiver” (MH3) and wanting that person to be “a happy patient” (MH3). When asked to describe their main worry, males primarily commented about functional communication difficulties, saying things like: “My wife can’t articulate or speak…the past five or six years, I’ve been working with a mute” (MH4). Interestingly, we noted that when some males were given the opportunity to provide additional comments at the end of the survey and despite having provided functional descriptions across most of their previous answers, some ended the survey with quite emotive expressions of their burden. For example:

Communication, when the person is uptight and stressed and they can’t tell you what is wrong… [then in the additional comments section, this same carer wrote:]…caring for someone you love and have spent most of your life with has to be the most difficult situation for anyone providing care. It is totally emotionally draining. (MH16)

Another example was this male participant who initially described practical challenges around communication limitations: “Struggling to communicate. Lots of misunderstandings, lots of silences” (MH13) and then in the additional comments section this same participant wrote: “An unparalleled sense of ambiguous loss and agonising grief. I haven’t done lying, so I’ll say I’m okay” (MH13).

##### Females: Emotional burden

When asked to describe their main worry, most female participants - in comparison to males - answered this question with a more emotive tone than males, sharing rich accounts of the grief felt as a consequence of witnessing the progression of the disease in their loved ones. Females provided descriptions of emotional burden, for example: “I was frightened of dealing with the physical declines, but it’s the emotional loss that creates the despair” (FW20), while another wrote: “It is grieving for your partner while he is still there and changing into a different person that you don’t really want to be with. Good for feeling guilty, which you know doesn’t help” (FW75).

#### The absent son and exhausted daughter who is always ‘on-call’

##### The absent son

Three sons (2% of survey participants) took part in this study in comparison to 54 daughters. In addition to their very low participation, sons generally made short statements of facts and did not elaborate on their experience as carers. For example, when asked to describe their main worry in relation to their caring role, a son said: “he [father living with dementia] cannot problem solve” (MS3). All three sons had already accessed formal services and seemed willing to access more help, for example one said “is there more help out there?” (MS2) and another said: “Category 1 [Government funded carer package] does not support my father adequately… a quicker access to category 3, would mean less time dealing with cash flows when organising services to assist my father” (MS3). However, one son reacted quite strongly to the question asking about the circumstances or events which would make them consider placing their person with dementia into residential care. This participant said: “Never! I wouldn’t put my Dog in a Nursing Home” (MS1).

##### The always on-call daughter

Fifty-four survey participants (32%) identified as daughters. Most shared very strong opinions through their written comments. Many wrote about the service system being “broken” (FD16) and “painful to navigate” (FD23), particularly because most had other “extensive obligations” (FD16), leaving them with “literally no time” (FD16) to apply for services. One daughter said that her main worry was: “Having to always be ‘on call’… Not having any time to me” (FD14). Many commented about the need for more respite type services because the caring role seemed to negatively impact on their capacity to juggle caring for their own family (their own children) and caring for the person with dementia. Exemplified here:

Other than support workers, I don’t receive much support at all…Needing more time with my family that includes 2 teenage daughters…trying to balance my family as well as caring for my mum… I’m neglecting my own family a lot. Mum is very demanding of my time and needs lots of reassurance and help…It is a very difficult time and generally as a carer I feel quite isolated. I don’t connect with people as I don’t want to burden people with how I’m feeling about my carer role. (FD40)

##### A secondary carer

Daughters commented about having to spend increasingly more hours supporting a parent and/or both parents in some cases, saying that “current system/services, don’t address multiple caring” (FD16) and pointing to the “need for recognition that you, as a carer, could be responsible for two parents with dementia” (FD16). A daughter described herself as a “secondary carer” (FD44), with one of her parents being the primary carer, thus having to wait for the approval of that parent, to access formal services:

My father controls what happens…I am entirely without power…I know she and he need it, but he won't do it till the car crash happens…we are unlikely to get help because he has left it so late. I expect my mother to be incapacitated entirely before we get help. (FD44)

##### I am exhausted

Daughters conveyed a high degree of stress and anxiety, making comments like: “I am exhausted, I do not get a break, I am exhausted” (FD22) and others said:

I also need time for my appointments, dentist, hairdresser, doctors, etc. and when I have my fortnightly respite, it is sometimes not long enough as I have to fit EVERYTHING in on THAT DATE AND TIME. Which doesn’t always work AND the appointments are not always on time themselves and I spend my time worrying about being late. So, I don’t have much respite to relax. (FD1)

## Discussion

This research study aimed to identify gender differences in carers of people with dementia. While our statistical analysis of quantitative data failed to identify any significant gender differences, our qualitative analysis revealed strong themes where male carers of people with dementia approached the caring role differently to female carers, suggesting that purely quantitative surveys are likely not an appropriate methodology to capture gendered differences in how carers approach the caring role.

Males accounted for a small proportion (14%) of research participants in this study. Demographic estimates indicate that males account for at least 35% of carers of people with dementia in Australia, suggesting that males were underrepresented in this study (AIHW, 2012, 2021). The majority (92%) of males in this study identified as ‘a carer’, suggesting that those who did not take part in this study potentially did not identify with the “carer label” (Milligan & Morbey, 2013, p. 20), thus not identifying this research as relevant to their situation. Not identifying as ‘a carer’ could explain why males have a poor access to formal supports in comparison to female carers (Greenwood & Smith, 2015, p.163; Milligan & Morbey, 2013). Our findings reinforced previous studies suggesting that male carers of people with dementia are less likely to take part in research (Houde, 2002; Milligan & Morbey, 2013) and that the caring role might still be disproportionately falling on women (Alzheimer’s Association Report, 2016; Bott et al., 2017; Kasper et al., 2015).

The 2008-09 *Aged-Care Assessment Program* (ACAP) data indicated that sons accounted for 14% of carers of people with dementia who accessed ACAP services in Australia (AIHW, 2012). Yet in this study, only 2% of participants identified as sons, and from our cohort of participants who identified as an ‘adult child’ of the person with dementia, 95% identified as daughters. Sons were, therefore under-represented in this study, suggesting that sons might be less likely to take part in research in comparison to daughters and reinforcing previous studies which argued that daughters were more likely than sons to care for a parent (Bott et al., 2017; Kasper et al., 2015). This raises the question: why are sons - as an adult child of a person with dementia - not taking part in research investigating the experience of carers of people with dementia?

Consistent with previous research, sons in this study seemed to manage the caring role from a distance by delegating the care duties (mainly instrumental care needs) to formal services, and they also expressed being unsure of what support options were available (Grigorovich et al., 2016; Kokorelias et al., 2021). Our findings complement previous work investigating carer access to an Australian dementia peak body organisation, which suggested underutilisation by sons, representing only 4% of all primary carers who accessed those services, whilst daughters represented 22% of that cohort (Poisson et al., 2020). With greater gender equality in the workplace, traditional family roles will need to be reimagined and research suggests that more sons will soon need to take-up a primary carer role for a parent living with dementia, reinforcing the need for more research into the experience and needs of this specific cohort of carers of people with dementia (Grigorovich et al., 2016; McDonnell & Ryan, 2014; Milligan & Morbey, 2013; Sanders & McFarland, 2002).

Literature suggests that female carers experience significantly higher levels of carer burden in comparison to male carers (Brodaty et al., 2014; Myers et al., 2003; O’Rourke et al., 1996; Samulowitz et al., 2018; Zarit et al., 1986). In contrast, this study found male and female carers to report similar scores on the psychometric scales that measured carer burden (ZBI scores). Despite reporting similar levels of carer burden, male carers differed from female carers in the way they expressed their main concerns qualitatively. When asked to describe their main worry as caregivers, males typically provided brief responses – often only using one or two words and most commented about communication difficulties - whereas almost all females answered that same question with detailed emotive accounts of the impact of the caring role. However, we noticed that male carers did provide more detailed and emotive statements in response to the last question of the research survey, which explicitly prompted participants to share additional comments about their caregiver experience. Research suggests that males with traditional beliefs about masculinity tend to mask their emotions and feelings behind an apparent “emotional stoicism” (Addis & Mahalik, 2003, p.9; Mahalik et al., 1998) and focus on operationalising instrumental aspects of the caring role (Baker et al., 2010; Hellstrom et al., 2017), which could explain why male carers are perceived to express less carer burden and more resistance to help-seeking, in comparison to female carers (Berger et al., 2005; Poysti et al., 2012; Robinson et al., 2014, p.15). Our findings seem to fit with Connell’s theory of hegemonic masculinity (Connell, 1995; Connell & Messerschmidt, 2005) and O’Neil’s (1981a; 2008) Gender Role Conflict theory, which suggests that males have been socialised to adopt “restrictive gender-roles” (O’Neil, 2008, p.362) and to repress overt expressions of strain in order to protect their male identity (Fee et al., 2020, 2021; Mahalik et al., 1998), particularly while performing tasks historically perceived by society as “women’s work” (Calasanti & King, 2007, p.526; Miller & Kaufman, 1996). We suggest that to perform an appropriate assessment of burden in male carers, health professionals should encourage males to elaborate on their caregiver experience.

Additionally, research suggests that the impact of pre-existing family dynamics could explain why carers with similar burden scores might articulate different expressions of perceived burden during in-depth interviews (Snyder, 2000; Tatangelo et al., 2018). For example, pre-existing gender beliefs among family members, may explain imbalances in carer expectations, allocation of carer duties and help-seeking approach, particularly between sons and daughters (Addis & Mahalik, 2003; Tatangelo et al., 2018). Daughters are often expected to assume the caring role for their parent with dementia which might prevent them from readily seeking formal assistance (DiLeone, 2021; Romero-Moreno et al., 2014; Tatangelo et al., 2018), whereas sons will readily access formal help because they approach the caring role as “care managers” (Grigorovich et al., 2016, p.4) and may also receive more informal assistance, especially from their partners (Kwok, 2006; McDonnell & Ryan, 2013). Indeed, in this study, we found all sons had accessed formal services and expressed a desire for more support services.

We recommend that health professionals become mindful of this stereotypical male behaviour (Mahalik et al., 1998; Miller & Kaufman, 1996) – that is, a tendency to mask emotive expressions of burden behind descriptions of functional burden - when performing their clinical assessments with male carers, as otherwise clinicians might be misled by the face-value narratives offered by males and potentially fail to identify key areas of need. Health professionals should apply male-friendly interventions adapted to the way males approach the caring role and underpinned by norms of hegemonic masculinity (Addis & Mahalik, 2003; Malcher, 2005; Milligan & Morbey, 2013; Ricciardelli et al., 2012). For example, instead of applying emotion focused conversations (asking male carers about their feelings regarding the caring role), health professionals should consider that male carers are likely to treat the caregiving role as “a job” (Hellstrom et al., 2017, p.962) and therefore, apply task-focused conversations (asking male carers to describe how they are managing their caregiving responsibilities) (Greiger et al., 2015; McDonnell & Ryan, 2013; Robinson et al., 2014). Furthermore, research suggests that male carers may benefit from participating in skill-building psychoeducational group programs for men, advertised as “workshops” and focused on teaching practical strategies for managing specific tasks and/or concerns of the caring role (Lauderdale & Gallagher-Thompson, 2003, p. 65).

Our qualitative methods of enquiry showed that male carers expressed almost diametrically opposed needs to female carers. Males detailed clear preferences for practical home help/cleaning services, whilst showing a general disinterest in day respite types of services. In contrast, females showed a clear disinterest for home help/cleaning services but expressed a strong preference for respite services. Given that household cleaning duties have traditionally been performed by females and been perceived by society as feminine roles (Miller & Kaufman, 1996), one could speculate that male carers might be reluctant to perform such duties, because these might erode their sense of masculinity and also, because these would demand learning new skills at a time when they are experiencing high carer stress, thus increasing the likelihood of errors and failure (Fee et al., 2020; McDonnell & Ryan, 2013; Milligan & Morbey, 2013). The male approach to the caring role appears to be shaped by a need to minimise a sense of failure to protect their masculinity.

The findings also highlighted thematic differences between how male and female participants explained why they had not yet accessed help from formal services. Males said that they preferred to wait for their person with dementia to make that first step of asking for help. Male carers seemed to approach help-seeking by waiting for permission to take-over control of the situation, whereas female carers seemed more disease oriented in their decision-making process, waiting for symptom presentation to re-adjust their level of help-seeking. Our findings validate the perception that male carers will typically wait for a crisis before asking for help (McDonnell & Ryan, 2013; Milligan & Morbey, 2016).

Both genders expressed a strong aversion to residential care placement. However, gender differences emerged in the circumstances that would ultimately push carers to place their loved ones into care, with males contemplating placement only if their own health became at risk of being impacted by the caring role, whereas female carers identified physical aggression from their person with dementia as a deciding factor. Husbands/partners of the person with dementia represent 64% of male carers of people with dementia in Australia and they are mostly aged between 65-74 years (AIHW, 2012). Census data shows that 86% of males aged 65 and over, have a chronic disease and with increased age, males are at increased risk of coronary heart disease, frailty, and obesity, with coronary heart disease being the primary cause of death in Australian males (AIHW, 2019; Milligan & Morbey, 2016; Rodgers et al., 2019). We suggest that by virtue of their age and gender, male carers aged above 65 years are likely to experience a serious health event, thus likely to feel at risk, which could likely precipitate the institutionalisation of their person with dementia. This might explain why male carers of people with dementia have been reported to institutionalise their person with dementia sooner than female carers and why they have also been perceived to wait for a crisis before asking for help (Bartlett et al., 2018; Greenwood & Smith, 2015; McDonnell & Ryan, 2013; Schaffler-Schaden et al., 2021).

The (Alzheimer’s Disease International, 2019) identified that due to socially and culturally rooted norms which portray women as naturally inclined to be “better carers” than men (p.102), women often do not get to choose to become carers, thus putting women at a greater risk of poorer physical and mental health. The report advises that dementia care should not be limited to a “one-size-fits-all” approach and that “women cannot (and should not) shoulder the responsibility” of being the “de-facto carer” for a person with dementia (Alzheimer’s Disease International, 2019, p.103). This study, showed that male carers of people with dementia approach help-seeking differently to female carers, suggesting the need for the development of male-friendly support services, which could in-turn facilitate timely access to services by male carers and reduce the disproportionate dependence on female carers (Bott et al., 2017; Kasper et al., 2015).

## Limitations

This study recruited participants who had accessed the research webpage of an Australian dementia peak body organisation and/or who had been to information sessions run by that organisation, suggesting that our cohort was already looking for and/or accessing support services and therefore, may not be fully representative of the population of carers of people living with dementia. While there is potential that differently worded survey questions may have resulted in different answers, this exploratory study has provided a foundation from which future research should seek to further refine the needs of male carers and how best to address these needs in practice. There was a smaller sample of males in comparison to females, and even fewer sons, limiting the comprehensiveness of analyses pertaining specifically to the male carer experience. Future research should seek to include rigorous sample size estimations, aiming to recruit a sufficiently large sample of male carers, specifically including sons of people with dementia to better understand the needs of this cohort through exploratory qualitative research. This study was limited to a binary definition of gender, mostly looking at the differences between males and females, which could have excluded people with other gender identities. The survey also assumed that participants were able to read and understand English, which could have excluded people from culturally and linguistically diverse backgrounds.

## Conclusion

This research identified that male carers of people with dementia were less likely to take part in research and did approach the caring role differently to female carers. Whilst male carers experienced similar levels of carer burden to female carers and identified as ‘the carer’ in similar proportions to female carers, males adopted a functional approach to help-seeking, typically asking for practical domestic assistance and generally refraining from showing emotions. The male approach to the caring role might be restrained by socially constructed ideals of hegemonic masculinity (Connell, 1995; Connell & Messerschmidt, 2005; O’Neil, 1981b), explaining why male carers struggle to adapt to aspects of the caring role traditionally performed by females in a household (Milligan & Morbey, 2016). Male carers play an important role in supporting people with dementia and with increased dementia incidence, more males may soon need greater access to formal supports (Robinson et al., 2014). We propose that carer support services and healthcare agencies should consider that males will approach the caring role differently to females and therefore, these agencies should adapt their service offerings to better suit the approach and needs of male carers of people with dementia.

## Data Availability

The data that support the findings of this study are maintained in the University of New South Wales secured institutional data repository (UNSW OneDrive) and are available from the corresponding author (s) upon reasonable request.

## Appendix 1

### List of open-ended questions

#### I Am Currently Using Support Services

1. Which service has been the most useful for you, and why?
2. Which service has been the least useful for you, and why?
3. Do you feel that you receive enough support services? ☐ Yes ☐ No ☐ Unsure Please explain your answer to the question above:
4. What changes would you make to the services you receive (if any) to help them better meet your needs?

#### I Am Not Currently Accessing Support Services

5. Please explain why you do not use support services:
6. What changes could be made to services (if any) to help them better meet your needs?

#### All Participants are to Respond

7. What circumstances or events would trigger you to ask for help from services in order to better support the person with dementia?
8. Which circumstances or events (if any) would make you consider placing the person you support into a residential care facility for long-term care?
9. Please think of one problem (or main worry) you have encountered in the last six months in relation to your support role to the person with dementia. In a few words, please describe this problem or main worry below.
10. Do you have any other comments on your experiences of providing support to a person with dementia that you wish to share?

## References

[bibr1-14713012231201595] Addis M. E. Mahalik J. R. (2003). Men, masculinity, and the contexts of help seeking. American Psychologist, 58(1), 5–14. 10.1037/0003-066X.58.1.5 12674814

[bibr2-14713012231201595] Alzheimer’s Association Report . (2016). 2016 Alzheimer’s disease facts and figures. Alzheimer’s and Dementia, 12(4), 459–509. 10.1016/j.jalz.2016.03.001 27570871

[bibr4-14713012231201595] Alzheimer’s Disease International . (2019). World alzheimer report 2019: Attitudes to dementia. London: Alzheimer’s Disease International. https://www.alzint.org/resource/world-alzheimer-report-2019/

[bibr5-14713012231201595] Amirkhan J. H. (1990). A factor analytically derived measure of coping. Journal of Personality and Social Psychology, 59(5), 1066–1074. 10.1037/0022-3514.59.5.1066

[bibr6-14713012231201595] Amirkhan J. H. (1994). Criterion validity of a coping measure. Journal of Personality Assessment, 62(2), 242–261. 10.1207/s15327752jpa6202_6 8189334

[bibr7-14713012231201595] Australian Institute of Health and Welfare . (2012). Dementia in Australia 2012. AIHW. https://www.aihw.gov.au/reports/dementia/dementia-in-australia/summary

[bibr8-14713012231201595] Australian Institute of Health and Welfare . (2019). The health of Australia’s males. AIHW. https://www.aihw.gov.au/reports/men-women/male-health

[bibr9-14713012231201595] Australian Institute of Health and Welfare . (2021). Dementia in Australia. AIHW. https://www.aihw.gov.au/reports/dementia/dementia-in-aus

[bibr10-14713012231201595] Australian Institute of Health and Welfare . (2023). Dementia in Australia. AIHW*.* Retrieved from. https://www.aihw.gov.au/reports/dementia/dementia-in-aus

[bibr11-14713012231201595] Baker K. L. Robertson N. (2008). Coping with caring for someone with dementia: Reviewing the literature about men. Aging and Mental Health, 12(4), 413–422. 10.1080/13607860802224250 18791888

[bibr12-14713012231201595] Baker K. L. Robertson N. Connelly D. (2010). Men caring for wives or partners with dementia: Masculinity, strain and gain. Aging and Mental Health, 14(3), 319–327. 10.1080/13607860903228788 20425651

[bibr13-14713012231201595] Bartlett R. Gjernes T. Lotherington A. Obstefelder A. (2018). Gender, citizenship and dementia care: A scoping review of studies to inform policy and future research. Health and Social Care in the Community, 26(1), 14–26. 10.1111/hsc.12340 26990695

[bibr14-14713012231201595] Berger J. M. Levant R. McMillan K. K. Kelleher W. Sellers A. (2005). Impact of gender role conflict, traditional masculinity ideology, alexithymia, and age on men’s attitudes toward psychological help seeking. Psychology of Men and Masculinity, 6(1), 73–78. 10.1037/1524-9220.6.1.73

[bibr15-14713012231201595] Black H. K. Schwartz A. J. Caruso C. J. Hannum S. M. (2009). How personal control mediates suffering: Elderly husbands' narratives of caregiving. The Journal of Men’s Studies, 16(2), 177–192. 10.3149/jms.1602.177

[bibr16-14713012231201595] Bott N. T. Sheckter C. C. Milstein A. S. (2017). Dementia care, women's health, and gender equity: The value of well-timed caregiver support. JAMA Neurology, 74(7), 757–758. 10.1001/jamaneurol.2017.0403 28492832

[bibr17-14713012231201595] Braun V. Clarke V. (2006). Using thematic analysis in psychology. Qualitative Research in Psychology, 3(2), 77–101. 10.1191/1478088706qp063oa

[bibr18-14713012231201595] Braun V. Clarke V. (2021). One size fits all? What counts as quality practice in (reflexive) thematic analysis? Qualitative Research in Psychology, 18(3), 328–352. 10.1080/14780887.2020.1769238

[bibr20-14713012231201595] Brodaty H. Woodward M. Boundy K. Ames D. Balshaw R. (2014). Prevalence and predictors of burden in caregivers of people with dementia. American Journal of Geriatric Psychiatry, 22(8), 756–765. 10.1016/j.jagp.2013.05.004 24012226

[bibr21-14713012231201595] Calasanti T. King N. (2007). Taking ‘women's work’ ‘like a man’: Husbands' experiences of care work. The Gerontologist, 47(4), 516–527. 10.1093/geront/47.4.516 17766672

[bibr22-14713012231201595] Carers Australia N. S. W. (2016). Male care policy statement. Carers Australia NSW. http://mengage.org.au/images/social/2017-06-16-Male-Carer-Policy-Statement.pdf

[bibr25-14713012231201595] Connell R. W. (1995). Masculinities. University of California Press.

[bibr26-14713012231201595] Connell R. W. Messerschmidt J. W. (2005). Hegemonic masculinity: Rethinking the concept. Gender & Society, 19(6), 829–859. 10.1177/0891243205278639

[bibr93-14713012231201595] Creswell J. W. Plano Clark V. L. (2006). Designing and Conducting Mixed Methods Research. Thousand Oaks, CA: Sage.

[bibr27-14713012231201595] Dam A. E. H. van Boxtel M. P. J. Rozendaal N. Verhey F. R. J. de Vugt M. E. (2017). Development and feasibility of Inlife: A pilot study of an online social support intervention for informal caregivers of people with dementia. PLoS One, 12(9), Article e0183386. 10.1371/journal.pone.0183386 28886056 PMC5590823

[bibr29-14713012231201595] Dementia Australia . (2023). Dementia Statistics: Key facts and statistics. Dementia Australia. https://www.dementia.org.au/statistics

[bibr30-14713012231201595] Desmond D. M. Shevlin M. MacLachlan M. (2006). Dimensional analysis of the coping strategy indicator in a sample of elderly veterans with acquired limb amputations. Personality and Individual Differences, 40(2), 249–259. 10.1016/j.paid.2005.04.015

[bibr31-14713012231201595] DiLeone C. (2021). Experiences of daughters caring for a parent with alzheimer's disease living at home. Research in Gerontological Nursing, 14(4), 191–199. 10.3928/19404921-20210428-02 34288784

[bibr32-14713012231201595] Fee A. McIlfatrick S. Ryan A. (2020). Examining the support needs of older male spousal caregivers of people with a long‐term condition: A systematic review of the literature. International Journal of Older People Nursing, 15(3), Article e12318. 10.1111/opn.12318 32367662

[bibr33-14713012231201595] Fee A. McIlfatrick S. Ryan A. (2021). ‘When it faded in her it faded in me’: A qualitative study exploring the impact of care-giving on the experience of spousal intimacy for older male care-givers. Ageing and Society, 41(1), 29–50. 10.1017/S0144686X19000850

[bibr34-14713012231201595] Geiger J. R. Wilks S. E. Lovelace L. L. Chen Z. Spivey C. A. (2015). Burden among male Alzheimer’s caregivers. American Journal of Alzheimer’s Disease and Other Dementias, 30(3), 238–246. 10.1177/1533317514552666 PMC10852689 25267930

[bibr35-14713012231201595] Greenwood N. Smith R. (2015). Barriers and facilitators for male carers in accessing formal and informal support: A systematic review. Maturitas, 82(2), 162–169. 10.1016/j.maturitas.2015.07.013 26271710

[bibr36-14713012231201595] Grigorovich A. Rittenberg N. Dick T. McCann A. Abbott A. Kmielauskas A. Estioko V. Kulasingham S. Cameron J. I. (2016). Roles and coping strategies of sons caring for a parent with dementia. American Journal of Occupational Therapy, 70(1), 1–9. 10.5014/ajot.2016.017715 26709428

[bibr37-14713012231201595] Hayes J. Zimmerman M. K. Boylstein C. (2010). Responding to symptoms of Alzheimer’s disease: Husbands, wives, and the gendered dynamics of recognition and disclosure. Qualitative Health Research, 20(8), 1101–1115. 10.1177/1049732310369559 20448273

[bibr38-14713012231201595] Hellström I. Håkanson C. Eriksson H. Sandberg J. (2017). Development of older men’s caregiving roles for wives with dementia. Scandinavian Journal of Caring Sciences, 31(4), 957–964. 10.1111/scs.12419 28124456

[bibr39-14713012231201595] Herreen D. Rice S. Currier D. Schlichthorst M. Zajac I. (2021). Associations between conformity to masculine norms and depression: Age effects from a population study of Australian men. BMC Psychology, 9(1), 32–32. 10.1186/s40359-021-00533-6 33608063 PMC7893732

[bibr40-14713012231201595] Holm S. (1979). A simple sequentially rejective multiple test procedure. Scandinavian Journal of Statistics, 6(2), 65–70. http://www.jstor.org/stable/4615733

[bibr41-14713012231201595] Houde S. C. (2002). Methodological issues in male caregiver research: An integrative review of the literature. Journal of Advanced Nursing, 40(6), 626–640. 10.1046/j.1365-2648.2002.02423.x 12473041

[bibr42-14713012231201595] Kasper J. D. Freedman V. A. Spillman B. C. Wolff J. L. (2015). The disproportionate impact of dementia on family and unpaid caregiving to older adults. Health Affairs, 34(10), 1642–1649. 10.1377/hlthaff.2015.0536 26438739 PMC4635557

[bibr43-14713012231201595] Kokorelias K. M. Naglie G. Gignac M. A. Rittenberg N. Cameron J. I. (2021). A qualitative exploration of how gender and relationship shape family caregivers’ experiences across the Alzheimer’s disease trajectory. Dementia, 20(8), 2851–2866. 10.1177/14713012211019502 33998323 PMC8678646

[bibr44-14713012231201595] Kwok H. (2006). The son also acts as major caregiver to elderly parents. Current Sociology, 54(2), 257–272. 10.1177/0011392106056745

[bibr45-14713012231201595] Lauderdale S. A. Gallagher-Thompson D. (2003). Men providing care. Clinical Gerontologist, 26(1-2), 53–70. 10.1300/J018v26n01_06

[bibr46-14713012231201595] Liamputtong P. (2020). Qualitative research methods (5th ed.). Oxford University Press.

[bibr47-14713012231201595] Lin C.-Y. Ku L.-J. E. Pakpour A. H. (2017). Measurement invariance across educational levels and gender in 12-item Zarit Burden Interview (ZBI) on caregivers of people with dementia. International Psychogeriatrics, 29(11), 1841–1848. 10.1017/S1041610217001417 28760167

[bibr48-14713012231201595] Maguire M. Delahunt B. (2017). Doing a thematic analysis: A practical, step-by-step guide for learning and teaching scholars. AISHE-J, 8(3), 3351–33514. http://ojs.aishe.org/index.php/aishe-j/article/view/3354

[bibr49-14713012231201595] Mahalik J. R. Cournoyer R. J. DeFranc W. Cherry M. Napolitano J. M. (1998). Men’s gender role conflict and use of psychological defenses. Journal of Counseling Psychology, 45(3), 247–255. 10.1037/0022-0167.45.3.247

[bibr50-14713012231201595] Malcher G. (2005). Men’s health, GPs, and ‘GPs4Men’ Australian Family Physician, 34(1-2), 21–23. 10.3316/informit.370187907790814 15727353

[bibr51-14713012231201595] McDonnell E. Ryan A. (2013). Male caregiving in dementia: A review and commentary. Dementia, 12(2), 238–250. 10.1177/1471301211421235 24336771

[bibr52-14713012231201595] McDonnell E. Ryan A. A. (2014). The experience of sons caring for a parent with dementia. Dementia, 13(6), 788–802. 10.1177/1471301213485374 24339083

[bibr53-14713012231201595] McKenzie S. K. Collings S. Jenkin G. River J. (2018). Masculinity, social connectedness, and mental health: Men’s diverse patterns of practice. American Journal of Men’s Health, 12(5), 1247–1261. 10.1177/1557988318772732 PMC6142169 29708008

[bibr54-14713012231201595] Miller B. Kaufman J. E. (1996). Beyond gender stereotypes: Spouse caregivers of persons with Dementia. Journal of Aging Studies, 10(3), 189–204. 10.1016/S0890-4065(96)90020-1

[bibr55-14713012231201595] Milligan C. Morbey H. (2013). Older men who care: Understanding their support and support needs. Lancaster University Centre for Ageing Research. https://eprints.lancs.ac.uk/68443/1/Older_men_who_care_report_2013Final.pdf

[bibr56-14713012231201595] Milligan C. Morbey H. (2016). Care, coping and identity: Older men’s experiences of spousal care-giving. Journal of Aging Studies, 38(2016), 105–114. 10.1016/j.jaging.2016.05.002 27531457

[bibr57-14713012231201595] Myers C. D. Riley J. L. Robinson M. E. (2003). Psychosocial contributions to sex- correlated differences in pain. The Clinical Journal of Pain, 19(4), 225–232. 10.1097/00002508-200307000-00005 12840616

[bibr59-14713012231201595] Nowell L. S. Norris J. M. White D. E. Moules N. J. (2017). Thematic analysis. International Journal of Qualitative Methods, 16(1), 1–13. 10.1177/1609406917733847

[bibr60-14713012231201595] O'Neil J. M. (1981a). Male sex role conflicts, sexism, and masculinity: Psychological implications for men, women, and the counseling psychologist. The Counseling Psychologist, 9(2), 61–80. 10.1177/001100008100900213

[bibr61-14713012231201595] O’Neil J. M. (1981b). Patterns of gender role conflict and strain: Sexism and fear of femininity in men’s lives. Personnel and Guidance Journal, 60(4), 203–210. 10.1002/j.2164-4918.1981.tb00282.x

[bibr62-14713012231201595] O'Neil J. M. (2008). Summarizing 25 Years of research on men's gender role conflict using the gender role conflict scale. The Counseling Psychologist, 36(3), 358–445. 10.1177/0011000008317057

[bibr63-14713012231201595] O’Rourke N. Haverkamp B. E. Rae S. Tuokko H. Hayden S. Beattie B. L. (1996). Response biases as a confound to expressed burden among spousal caregivers of suspected dementia patients. Psychology and Aging, 11(2), 377–380. 10.1037/0882-7974.11.2.377 8795067

[bibr65-14713012231201595] Poisson V. O. Withall A. Reilly A. Poulos R. G. (2020). Are male carers of people with dementia underutilising support services? Australasian Journal on Ageing, 39(4), 389–390. 10.1111/ajag.12889 33377294

[bibr66-14713012231201595] Pöysti M. M. Laakkonen M.-L. Strandberg T. Savikko N. Tilvis R. S. Eloniemi- Sulkava U. Pitkälä K. H. (2012). Gender differences in dementia spousal caregiving. International Journal of Alzheimer’s Disease, 2012, 162960–162965, 162960. 10.1155/2012/162960 PMC3465980 23056990

[bibr67-14713012231201595] Raifman S. DeVost M. A. Digitale J. C. Chen Y.-H. Morris M. D. (2022). Respondent- driven sampling: A sampling method for hard-to-reach populations and beyond. Current Epidemiology Report, 9(2022), 38–47. 10.1007/s40471-022-00287-8

[bibr68-14713012231201595] Ricciardelli L. Mellor D. McCabe M. (2012). The quiet crisis: Challenge for men’s health in Australia. InPsych, 34(4). https://psychology.org.au/inpsych/2012/august/ricciardelli#

[bibr69-14713012231201595] Robinson C. A. Bottorff J. L. Pesut B. Oliffe J. L. Tomlinson J. (2014). The male face of caregiving. American Journal of Men's Health, 8(5), 409–426. 10.1177/1557988313519671 24414033

[bibr70-14713012231201595] Rodgers J. L. Jones J. Bolleddu S. I. Vanthenapalli S. Rodgers L. E. Shah K. Karia K. Panguluri S. K. (2019). Cardiovascular risks associated with gender and aging. Journal of cardiovascular development and disease, 6(2), 19. 10.3390/jcdd6020019 31035613 PMC6616540

[bibr71-14713012231201595] Romero-Moreno R. Losada A. Marquez M. Laidlaw K. Fernandez- Fernandez V. Nogales-Gonzalez C. Lopez J. (2014). Leisure, gender, and kinship in dementia caregiving: Psychological vulnerability of caregiving daughters with feelings of guilt. Journals of Gerontology Series B: Psychological Sciences and Social Sciences, 69(4), 502–513. 10.1093/geronb/gbt027 23685923

[bibr72-14713012231201595] Russell R. (2001). In sickness and in health: A qualitative study of elderly men who care for wives with dementia. Journal of Aging Studies, 15(4), 351–367. 10.1016/S0890-4065(01)00028-7

[bibr73-14713012231201595] Samulowitz A. Gremyr I. Eriksson E. Hensing G. (2018). “Brave men” and “emotional women”: A theory-guided literature review on gender bias in health care and gendered norms towards patients with chronic pain (2018). Pain research and management, 6358624. 10.1155/2018/6358624 PMC5845507 29682130

[bibr75-14713012231201595] Sanders S. McFarland P. (2002). Perceptions of caregiving role by son’s caring for a parent with alzheimer’s disease. Journal of Gerontological Social Work, 37(2), 61–76. 10.1300/J083v37n02_06

[bibr76-14713012231201595] Schaffler-Schaden D. Krutter S. Seymer A. Eßl-Maurer R. Flamm M. Osterbrink J. (2021). Caring for a relative with dementia: Determinants and gender differences of caregiver burden in the rural setting. Brain Sciences, 11(11), 1511. 10.3390/brainsci11111511 34827510 PMC8615550

[bibr77-14713012231201595] Sharma N. Chakrabarti S. Grover S. (2016). Gender differences in caregiving among family – caregivers of people with mental illnesses. World Journal of Psychiatry, 6(1), 7–17. 10.5498/wjp.v6.i1.7 27014594 PMC4804270

[bibr78-14713012231201595] Snyder J. R. (2000). Impact of caregiver-receiver relationship quality on burden and satisfaction. Journal of Women and Aging, 12(1-2), 147–167. 10.1300/J074v12n01_10 10986856

[bibr79-14713012231201595] Tatangelo G. McCabe M. Macleod A. Konis A. (2018). I just can't please them all and stay sane: Adult child caregivers’ experiences of family dynamics in care‐giving for a parent with dementia in Australia. Health and Social Care in the Community, 26(3), e370–e377. 10.1111/hsc.12534 29322577

[bibr81-14713012231201595] Zarit S. H. Todd P. A. Zarit J. M. (1986). Subjective burden of husbands and wives as caregivers: A longitudinal study. The Gerontologist, 26(3), 260–266. 10.1093/geront/26.3.260 3721233

